# Construction and Comparison of UPLC-QE-Orbitrap-MS and UPLC-MS/MS Methods for the Detection of Diazepam Residues in Complex Aquatic Matrices

**DOI:** 10.3390/foods14193296

**Published:** 2025-09-23

**Authors:** Yuanhao Yang, Yi Li, Qiangbing Tian, Juanning Yang, Yiming Xu, Wen Su, Fei Wang, Siyao Qu, Sien Wen, Wei Cao

**Affiliations:** 1 College of Food Science and Technology, Northwest University, Xi’an 710069, China; yuanhao_y@126.com; 2 Fisheries Research and Technology Extension Center of Shaanxi, Xi’an 710086, China; 18202989150@163.com (Y.L.); qiangbingtian@sina.com (Q.T.); yjn2005@sohu.com (J.Y.); zmjsxym@alu.cau.edu.cn (Y.X.); nm1876171@163.com (F.W.); 15399302508@189.cn (S.Q.); wensien@sohu.com (S.W.); 3 Fishery Environment and Aquatic Products Quality Inspection-Testing Center, Ministry of Agriculture and Rural Affairs Xi’an, Xi’an 710086, China; 4 School of Bioscience and Engineering, Shaanxi University of Technology, Hanzhong 723001, China; 15319384579@163.com

**Keywords:** diazepam metabolites, complex aquatic matrices, ultra-performance liquid chromatography-orbitrap MS, high-resolution MS, tandem triple quadrupole MS

## Abstract

A standardized pretreatment protocol was established for simultaneous determination of diazepam and its metabolites—nordazepam, oxazepam, and temazepam—in aquatic products using liquid chromatography–mass spectrometry. Samples were extracted with 0.2% formic acid in acetonitrile (solid–liquid ratio 1:5, *m*/*v*), purified via MCX solid-phase extraction, eluted with 5% ammoniated methanol, and concentrated under reduced pressure. The residue was reconstituted in 0.2% formic acid–50% acetonitrile aqueous solution. Chromatographic and mass spectrometric conditions were optimized on two platforms: UPLC-QE-Orbitrap-MS and UPLC-MS/MS, with quantification based on internal standards. Both platforms showed excellent linearity across 0.2–200 ng/mL (R^2^ > 0.997), with detection and quantification limits as low as 0.1 μg/kg and 0.2 μg/kg, respectively. Following Codex Alimentarius guidelines (CAC/GL-71), 330 matrix samples (intra-batch *n* = 6, inter-batch *n* = 5) were validated, showing strong inter-platform agreement (Pearson r > 0.990, *p* < 0.001). Intra-batch RSDs ranged from 1.86% to 14.64%, and inter-batch RSDs from 1.10% to 11.41%. Recoveries ranged from 73.8% to 117.9% (*p* > 0.05). The dual-platform detection system developed herein demonstrates high sensitivity, strong matrix interference resistance, and excellent reproducibility, enabling accurate trace quantification of diazepam and its metabolites in heterogeneous aquatic samples.

## 1. Introduction

Psychoactive drugs in the environment, as a new class of environmental persistent pharmaceutical pollutants (EPPPs), have drawn global attention in environmental science and public health due to their potential ecological risks. These substances may exhibit endocrine-disrupting properties and cause genetic, developmental, and immune effects, contributing to the emergence of antibiotic-resistant microorganisms. They pose serious potential threats to human health and ecological environments. Numerous studies have reported the presence of drugs such as morphine and cocaine in surface waters and wastewater across multiple countries, underscoring the need for further research into their environmental fate, transformation, and ecological effects [1,2,3,4].

Among these, diazepam (DZP), a widely used benzodiazepine anxiolytic, stands out due to its extensive use and environmental persistence. As a typical EPPP, diazepam is highly resistant to degradation, ubiquitously detected in aquatic environments, and capable of long-term persistence [5,6]. Beyond medical applications, diazepam is also frequently employed in aquaculture to reduce stress, enhance feed efficiency, and promote growth [7]. For instance, supplementation of aquatic feed with 12.0 mg/100 g of diazepam significantly improved feed conversion in Nile tilapia by enhancing GABAergic signaling and suppressing serotonin receptor activity in the hypothalamic satiety center [8].

However, the widespread use of diazepam has led to serious environmental contamination. Diazepam enters aquatic environments through various pathways, including domestic sewage, medical wastewater, and aquaculture effluent. It then undergoes a chain reaction of “migration and diffusion—adsorption and retention—transformation and degradation—desorption and release—bioaccumulation,” directly threatening the survival and reproduction of aquatic organisms. Furthermore, through bioaccumulation and transmission along the food chain, it may pose risks to human health [9]. Its environmental risk is further compounded by its chemical stability and metabolic pathways: diazepam resists photolytic degradation and undergoes biotransformation via demethylation and C-3 oxidation to generate active metabolites, including nordazepam, temazepam, and subsequently oxazepam [10]. These metabolites retain pharmacological activity [11], resulting in complex bioaccumulation dynamics and transformation cascades that contribute to sustained ecological exposure. Although diazepam residues have been detected in various aquatic products [12,13,14], their metabolic enrichment, toxicological effects, and dietary exposure risks remain inadequately characterized [15].

Consequently, there is an urgent need for robust analytical methods capable of simultaneously detecting diazepam and its metabolites with high sensitivity and precision in diverse aquatic matrices. Existing detection technologies face multiple limitations. GC-MS typically requires derivatization and offers limited sensitivity, with LOQs around 2.0 μg/kg [16]. High-performance liquid chromatography (HPLC) is prone to matrix interference, resulting in false positives and reduced sensitivity [17]. Although ultrahigh-performance liquid chromatography-tandem mass spectrometry (UPLC-MS/MS) has been applied to samples from animals, feed, and forensic investigations [18,19,20,21], it performs poorly in complex aquatic matrices, with LODs and LOQs typically ranging between 0.5 and 1.0 μg/kg and 1.0–2.5 μg/kg, respectively [13,14,22], falling short for trace-level analyses.

Compounding these challenges is the considerable heterogeneity in matrix composition across different aquatic species (e.g., fish, shrimp, shellfish, and crabs), with varying contents of endogenous substances such as proteins and minerals [23,24]. This matrix complexity severely impairs mass spectrometric performance and is difficult to accurately correct using conventional approaches [24]. As a result, existing methods often suffer from limited sensitivity, poor coverage, and inadequate reproducibility when detecting multiple analytes simultaneously in aquatic samples—limitations that are especially problematic for toxicological assessments and food safety monitoring. While UPLC-MS/MS has gained traction for evaluating emerging pollutants in aquatic ecosystems [25], its application in metabolomic profiling of sedative residues remains underexplored, necessitating focused methodological development.

This study aims to address these gaps by developing a high-sensitivity, robust detection method for diazepam and its metabolites, specifically tailored for complex aquatic matrices. To achieve this, the edible tissues of five representative aquatic species—large yellow croaker (bony fish), Litopenaeus vannamei (crustacean), bay scallop (bivalve), three-wart crab (crustacean), and squid (cephalopod)—were used to construct a comprehensive matrix model. A standardized pretreatment procedure was established through systematic optimization of sample preparation, chromatographic separation, and mass spectrometric conditions. The analytical performance of UPLC-QE-Orbitrap-MS and UPLC-MS/MS platforms was then evaluated in parallel, enabling cross-validation of results.

This study overcomes key limitations in current detection strategies by significantly improving sensitivity, matrix tolerance, and methodological reliability. The dual-platform system provides a powerful analytical tool for accurately quantifying diazepam and its metabolites in complex biological samples, offering critical technical support for ecological risk assessment, aquatic food safety, and public health protection.

## 2. Materials and Methods

### 2.1. Instruments and Reagents

Analytical instrumentation included a Q-Exactive Quadrupole/Orbitrap High-Resolution Mass Spectrometer (Thermo Fisher Scientific, Waltham, MA, USA) and a QTRAP 5500 Tandem Triple Quadrupole Mass Spectrometer (AB Sciex, Redwood City, CA, USA). Supporting equipment included a BP211D analytical balance (Sartorius AG, Goettingen, Germany), an ME1002T electronic balance (METTLER TOLEDO, Shanghai, China), an RE52AA rotary evaporator (Shanghai Yarong Biochemical Instrument Factory, Shanghai, China), an LXJ-II.B centrifuge (Shanghai Anting Scientific Instrument Factory), and a Sorvall-Stratos refrigerated centrifuge (Thermo Fisher Scientific, USA).

Analytical standards for diazepam, nordazepam, oxazepam, and temazepam (1000 μg/mL in methanol) were obtained from a U.S.-based supplier (Stanford Chemicals Company, Lake Forest, CA, USA). Isotope-labeled internal standards (D5-diazepam, D5-nordazepam, D5-oxazepam, and D5-temazepam) were prepared at 100 μg/mL in methanol (Supplier: Stanford Chemical Company). LC-MS grade acetonitrile, methanol, and formic acid were procured from Shanghai Anpu Experimental Technology Co., Ltd. (ANPEL, Shanghai, China). Analytical-grade ammonia was supplied by Sinopharm Chemical Reagent Co., Ltd. (Shanghai, China), and Wahaha ultrapure water was used throughout. SelectCore MCX solid-phase extraction cartridges (200 mg/6 mL) were purchased from Suzhou Napu Analytical Technology Co., Ltd. (NanoChrom, Suzhou, China).

### 2.2. Sample Preparation

#### 2.2.1. Extraction Procedure

A 2.00 g aliquot of homogenized tissue (±0.05 g) was weighed into a 50 mL centrifuge tube. Then, 40 μL of mixed internal standard working solution was added, followed by 5 mL of 0.1% formic acid in 70% acetonitrile. The mixture was vortexed for 5 min, sonicated for 5 min, and centrifuged at 5000 rpm for 10 min. The supernatant was transferred to a new tube, and the extraction was repeated once under the same conditions. The combined extracts were diluted with 10 mL of water, mixed thoroughly, and subjected to purification.

#### 2.2.2. Solid-Phase Extraction (SPE)

SelectCore MCX is a mixed-mode strong cation exchange solid-phase extraction column based on monodisperse polymer microspheres. It has good water wettability and chemical stability and has good retention for both strong and weak basic compounds. The MCX cartridges were conditioned with 5 mL of methanol and 5 mL of 1% formic acid aqueous solution. The sample extract was loaded onto the cartridge. After complete flow-through, the cartridge was sequentially washed with 10 mL of 1% formic acid aqueous solution and 10 mL of 1% formic acid–acetonitrile solution. Elution was performed with 10 mL of 5% ammoniated methanol, and the eluate was collected in a 50 mL round-bottom flask. The eluate was evaporated to near dryness under reduced pressure at 35 °C, redissolved in 1.00 mL of 0.2% formic acid in 50% acetonitrile, filtered through a 0.22 μm membrane, and analyzed by LC-MS.

### 2.3. Standard Solution Preparation

#### 2.3.1. Mixed Standard Stock Solution

Appropriate volumes of diazepam, nordazepam, oxazepam, and temazepam standards were diluted with methanol to prepare a mixed stock solution at a concentration of 50.0 ng/mL. The solution was stored at −18 °C.

#### 2.3.2. Mixed Internal Standard Stock Solution

Isotope-labeled internal standards were diluted with methanol to a final concentration of 100 ng/mL and stored at 4 °C.

### 2.4. Instrumental Conditions

#### 2.4.1. UPLC-QE-Orbitrap-MS Conditions

##### Chromatographic Conditions

Separation was performed using a HSS T3 column (Waters, Milford, MA) (2.1 mm × 100 mm, 1.7 μm) at 35 °C. The injection volume was 10 μL, and the flow rate was 0.30 mL/min. The mobile phases consisted of 0.2% formic acid in water (A) and methanol (B). Gradient elution was programmed as follows:

0–1.0 min: 15% B; 1.0–2.5 min: 15–85% B; 2.5–3.5 min: 85% B; 3.5–4.5 min: 85–15% B; 4.5–5.5 min: 15% B.

##### Mass Spectrometry Conditions

High-resolution detection was performed on the Q Exactive Orbitrap mass spectrometer using an HESI-II source in positive ion mode. Acquisition was conducted in parallel reaction monitoring (PRM) mode. Source parameters were spray voltage 3.5 kV, lens voltage 55 V, capillary temperature 325 °C, auxiliary gas heater temperature 325 °C, sheath gas at 40 arbitrary units (4 × 10^7^ Pa), and auxiliary gas at 10 arbitrary units (1 × 10^7^ Pa). The resolution was set to 70,000 (FWHM). The AGC target was 3 × 10^6^, with a maximum injection time of 200 ms and a maximum dwell time of 50 ms.

#### 2.4.2. UPLC-MS/MS Conditions

##### Chromatographic Conditions

Chromatographic separation was carried out on a C18 column (100 mm × 2.1 mm, 3 μm) at 35 °C, with an injection volume of 10 μL and a flow rate of 0.30 mL/min. The mobile phases were 0.2% formic acid in water (A) and methanol (B), with a gradient as follows: 0–2.5 min: 5% B; 2.5–3.0 min: 5–95% B; 3.0–5.0 min: 95% B; 5.0–5.5 min: 95–5% B; 5.5–8.0 min: 5% B.

##### Mass Spectrometry Conditions

Mass detection was performed using an ESI source in positive ion mode. Source temperature was 550 °C, spray voltage 5500 V, nebulizer gas pressure 55 psi, auxiliary gas pressure 55 psi, and curtain gas pressure 35 psi. Monitoring was performed using multiple reaction monitoring (MRM). Q1 and Q3 resolutions were set to 0.7 Da (FWHM).

#### 2.4.3. Qualitative Confirmation

Retention times of analytes in test samples were required to match those of reference standards within ±2.5% under identical conditions. Fragment ion relative abundances were compared with calibration standards at equivalent concentrations. Consistency in the abundance ratio of the base peak to the next most intense ion was assessed according to EU guideline 2002/657/EC [26].

#### 2.4.4. Quantitative Analysis

Quantitation was based on internal standard calibration using a 7-point standard curve, with concentration levels selected to match sample analyte content. All analyte responses had to fall within the instrument’s linear range. Samples exceeding this range were diluted with the appropriate solvent prior to analysis.

### 2.5. Subsection

Method accuracy and precision were evaluated through spiked recovery experiments at three concentration levels using blank matrix samples. Intra-batch repeatability was assessed with six replicates per level, while inter-batch precision was determined by analyzing five replicates over five consecutive weeks.

### 2.6. Matrix Model Construction

A representative matrix spectrum was established using five common aquatic species spanning key taxonomic groups: large yellow croaker (*Larimichthys crocea*, bony fish), Pacific white shrimp (*Litopenaeus vannamei*, crustacean), bay scallop (*Argopecten irradians*, mollusk), three-wart crab (*Portunus trituberculatus*, crustacean), and squid (*Teuthida*, cephalopod).

### 2.7. Statistical Analysis

Pearson correlation analysis and paired *t*-tests were employed to compare the detection results of diazepam and its three metabolites between the two mass spectrometry platforms, respectively. In this study, five different biological matrices were used as the research objects, and three concentration levels (0.2, 0.4, and 10.0 µg/kg) were added to each matrix for detection. By calculating the covariance and standard deviation of the samples, the Pearson correlation coefficients (r) of the detection results of diazepam, nordazepam, oxazepam, and temazepam between the two platforms could be obtained; when r = 1, a perfect linear positive correlation was observed between the two sets of results. Meanwhile, since the same batch of samples was measured using the two detection platforms in this study, the data exhibited a paired relationship, which met the conditions for the paired *t*-test. Therefore, this test could be used to evaluate the differences in detection results, thereby verifying the stability of the detection results of the two platforms.

## 3. Results and Discussion

### 3.1. Optimization of Sample Preparation Conditions

#### 3.1.1. Selection of Extraction Solvents

Diazepam and its metabolites are weakly polar compounds, soluble in organic solvents such as methanol, acetonitrile, and ethyl acetate, but poorly soluble in water. Given the high lipid and protein content in aquatic matrices, acetonitrile was chosen as the extraction solvent due to its broad polarity range, low molecular weight, and strong tissue penetration. It effectively precipitates proteins and reduces the co-extraction of lipophilic and nonpolar interferences commonly associated with medium-polarity solvents [27].

In alignment with the properties of the MCX solid-phase extraction (SPE) cartridge used later in the purification stage, an acidified acetonitrile-water solution was adopted. To enhance extraction efficiency, we evaluated the effects of formic acid concentrations (0.05%, 0.1%, 0.5%, and 1% *v*/*v*) and acetonitrile proportions (50%, 60%, 70%, and 80% *v*/*v*) on recoveries of the four target analytes. Considering both extraction efficiency and matrix effects, the optimal extraction solvent was determined to be 0.1% formic acid in 70% acetonitrile-water.

**Figure 1:**
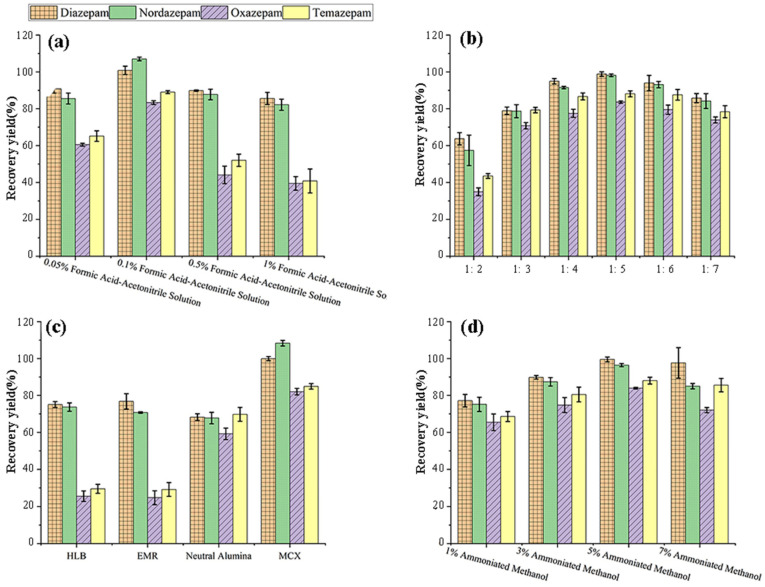
(**a**). Effect of formic acid concentration in the extraction solvent on extraction efficiency for the target analytes; (**b**). Effect of the solid–liquid ratio of sample mass to extraction solution volume on the extraction efficiency of target analytes; (**c**). Effect of alternative solid-phase extraction cleanup approaches on target analyte recovery; (**d**). Effect of varying ammoniated methanol proportions on the elution performance for the target analytes.

We also assessed the impact of the solid-to-liquid (S/L) ratio on extraction performance (Figure 1a,b). When the S/L ratio was ≤1:3, recoveries were significantly lower, likely due to insufficient solvent volume leading to incomplete extraction or increased matrix effects. Extraction efficiency improved with increasing solvent volume, peaking at an S/L ratio of 1:5. Ratios above this introduced more impurity peaks and prolonged purification. Thus, 1:5 was selected as the optimal S/L ratio to balance efficiency and practicality.

#### 3.1.2. Optimization of Purification Methods

##### Selection of Solid-Phase Extraction Columns

SPE is widely used to purify complex biological samples prior to analysis of drug residues. Four types of SPE cartridges were evaluated: Prime HLB (ANPEL, Shanghai, China) (200 mg/3 mL), Captiva EMR-Lipid (ANPEL, Shanghai, China) (300 mg/3 mL), neutral alumina (ANPEL, Shanghai, China) (500 mg/3 mL), and MCX (NanoChrom, Suzhou, China) (200 mg/6 mL). The effects of different purification methods on target compound recovery rates were compared. When using Prime HLB and EMR flow-through columns for purification, the recovery rates for all four target compounds were low, ranging from approximately 30–60%. Specifically, the recoveries for oxazepam and temazepam were only around 30%. This may be attributed to the competition for adsorption sites on the HLB column packing material by abundant fats and oils in aquatic products, leading to column overloading and reduced target compound recovery. Although EMR can remove lipid substances from the sample, it performs poorly against other polar or ionic interferents, resulting in mediocre purification of target compounds. When using neutral alumina columns for purification, the recovery rates for the four target compounds were relatively low, ranging from 60 to 70%. This is because neutral alumina lacks specificity in adsorbing target compounds, making it difficult to eliminate a large amount of impurity interference and leading to suboptimal purification results.

MCX columns, in contrast, delivered superior performance with recovery rates of 80–110% for all analytes and minimal impurity interference (Figure 1c). This was likely due to strong ionic interactions between the sulfonic acid groups on the cartridge and the analytes, which enhanced retention and selectivity. Based on these results, MCX cartridges were selected for all subsequent extractions.

##### Optimization of Elution Conditions

Diazepam and its metabolites are basic compounds containing nitrogen atoms. Adding alkaline agents to the eluent inhibits ionization, maintaining these compounds in their non-ionized (free base) form and enhancing elution. We tested methanol containing 1%, 3%, 5%, and 7% ammonia as eluents. As ammonia content increased, elution strength improved. Maximum recovery was achieved at 5% ammonia, likely because increased alkalinity suppressed protonation, promoting elution in molecular form. Above 5%, however, more impurity peaks emerged in chromatograms (Figure 1d). Consequently, 5% ammoniated methanol was selected as the optimal eluent.

### 3.2. Optimization of Chromatographic and Mass Spectrometric Conditions

#### 3.2.1. Optimization of Liquid Chromatography Conditions

**Figure 2:**
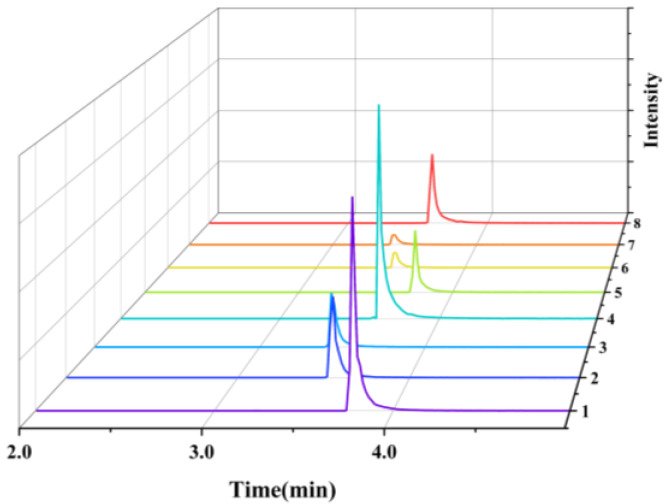
UPLC-QE-Orbitrap-MS Chromatograms and Retention Times (RT) of the Eight Target Compounds. 1: Diazepam (RT: 3.77); 2: Nordazepam (RT: 3.56); 3: Oxazepam (RT: 3.44); 4: Temazepam (RT: 3.63); 5: Diazepam-D5 (RT: 3.76); 6: Nordazepam-D5 (RT: 3.55); 7: Oxazepam-D5 (RT: 3.45); 8: Temazepam-D5 (RT: 3.62).

**Figure 3:**
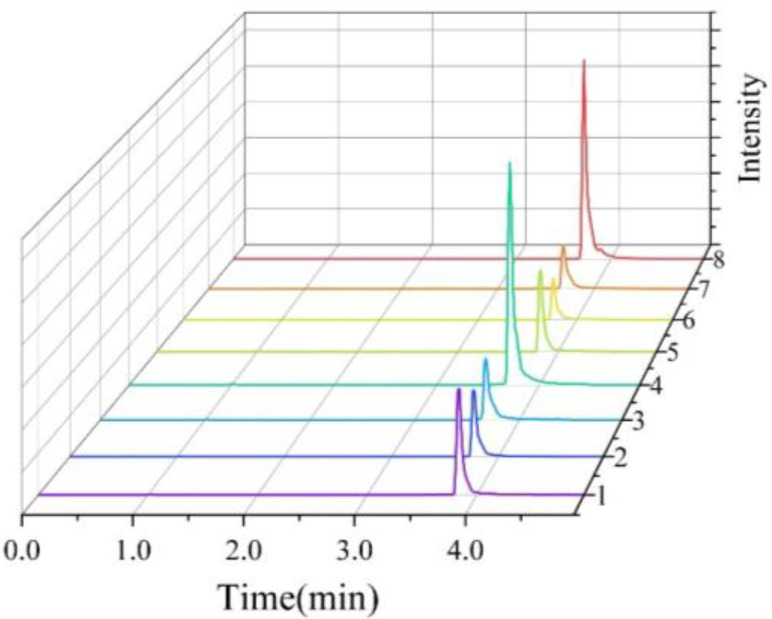
UPLC-MS/MS Chromatograms and Retention Times (RT) of the Eight Target Compounds. 1: Diazepam (RT: 3.84); 2: Nordazepam (RT: 3.76); 3: Oxazepam (RT: 3.68); 4: Temazepam (RT: 3.71); 5: Diazepam-D5 (RT: 3.82); 6: Nordazepam-D5 (RT: 3.76); 7: Oxazepam-D5 (RT: 3.68); 8: Temazepam-D5 (RT: 3.70).

Liquid chromatographic conditions were optimized using two platforms—UPLC-QE-Orbitrap-MS and UPLC-MS/MS—by evaluating mobile phase composition, column temperature, and flow rate. Six mobile phase systems were tested: water-acetonitrile, water-methanol, 0.1% and 0.2% formic acid with either water-acetonitrile or water-methanol. Column temperatures (25–40 °C) and flow rates (0.25–0.6 mL/min) were also assessed. Optimal results were achieved using 0.2% formic acid in water and methanol as the mobile phase, a flow rate of 0.30 mL/min, and a column temperature of 35 °C. Under these conditions, both instruments delivered high sensitivity, clean baselines, and excellent peak shapes, while effectively separating target compounds from matrix interferences (Figure 2 and Figure 3).

#### 3.2.2. Optimization of Mass Spectrometry Conditions

Given the basic nature of diazepam and its metabolites, which contain primary or tertiary amines, positive ionization was used. A heated electrospray ionization (HESI) source was employed on the UPLC-Q-Orbitrap-MS platform, and a standard electrospray ionization (ESI) source was used for UPLC-MS/MS.

For the UPLC-Q-Orbitrap-MS system, a Full MS-dd-MS^2^ acquisition mode was used. The protonated precursor ions were isolated by the quadrupole, fragmented via high-energy collision dissociation (HCD), and detected in the Orbitrap with high resolution. By optimizing collision energy, the fragment ion with the highest intensity was selected for quantification, while all fragments were used for qualitative analysis (Table 1). A precursor-to-fragment ion intensity ratio of approximately 3:1 was maintained for robust quantitation.

On the UPLC-MS/MS platform, standard syringe-pump infusion and precursor ion scanning in positive ESI mode were performed to identify characteristic product ions. For each compound, two highly responsive and low-interference fragment ions were chosen. Parameters such as declustering potential (DP) and collision energy (CE) were optimized accordingly (Table 2).

#### 3.2.3. Analysis of Mass Spectrometric Fragmentation Mechanisms

Using high-resolution accurate mass data from Q-Orbitrap-MS, the fragmentation pathways of diazepam, nordazepam, oxazepam, and temazepam were elucidated. The protonated molecules of diazepam ([M + H]^+^ *m*/*z* 285.07892, mass error: 0.35 ppm) and nordazepam ([M + H]^+^ *m*/*z* 271.06327, mass error: 0.37 ppm) undergo initial heterolytic cleavage of the amide bond, releasing CO, followed by azepine ring opening and subsequent elimination of benzonitrile (C_7_H_5_N), yielding stable fragment ions at *m*/*z* 154.04180 (mass error: 0.65 ppm) and 140.02615, respectively. An alternative fragmentation route involves the elimination of a chlorine radical (Cl·), forming odd-electron ions at *m*/*z* 222.11515 (mass error: 0.45 ppm) and 208.09950. Although this violates the classical even-electron rule, such pathways have been documented under high-energy collisions [28].

Additionally, diazepam exhibits a concerted cleavage of its diazepine ring—facilitated by the seven-membered lactam structure—resulting in the loss of methyl isocyanate (C_2_H_3_NO) and a subsequent chlorine radical to generate *m*/*z* 193.08860 (mass error: 0.51 ppm). For oxazepam ([M + H]^+^ *m*/*z* 287.05818, mass error: 0.69 ppm) and temazepam ([M + H]^+^ *m*/*z* 301.07383), fragmentation begins with proton migration and H_2_O elimination, forming *m*/*z* 269.04762 and 283.06327 (mass error: 0.38 ppm), respectively. These fragments further lose CO via amide bond cleavage, yielding ions at *m*/*z* 241.05270 (mass error: 1.24 ppm) and 255.06835.

Electrospray ionization (ESI), a soft ionization technique, typically promotes the loss of small neutral molecules, producing thermodynamically stable ions that are consistent with the minimum energy principle. The observed fragmentation pathways support the scientific rationale and validity of the applied technique. Notably, while Q-Orbitrap HRMS and QqQ MS display consistent fragmentation mechanisms, their fragment ion abundance profiles differ. Orbitrap HRMS, utilizing high-energy collision dissociation (HCD), often triggers multi-stage fragmentation, offering rich structural data and superior compound identification capabilities. In contrast, QqQ MS, through precise collision energy control, selectively enriches specific fragment ions, making it more effective for quantifying trace analytes.

In this study, common fragment ions from both platforms were selected as quantification ions. Structural confirmation leveraged Orbitrap HRMS’s accurate mass data, while QqQ MS’s high sensitivity enabled trace quantification. This dual-platform approach resulted in a robust qualitative-quantitative detection method characterized by high sensitivity, broad linearity, and strong anti-interference capability, offering valuable technical insight into the structural analysis and trace detection of sedative pharmaceuticals in complex biological matrices.

### 3.3. Methodological Investigation

#### 3.3.1. Matrix Effects and Their Elimination

Matrix effects (ME) are a common challenge in mass spectrometric analysis. This study employs the method adopted by Tomasz Tuzimski et al. [29] and the post-addition extraction method proposed by Cao Hui et al. [30] to quantitatively calculate ME, calculated as:

ME = (slope of the curve of peak area versus concentration for matrix − matched standard solution/slope of the curve of peak area versus concentration for matrix − free standard solution − 1) × 100%.

Using shrimp, scallop, swimming crab, and squid as matrices, the effect of adding internal standards corresponding to diazepam and its metabolites was assessed. Without correction, ME values ranged from 6.69% to 71.82%. After applying internal standards, ME values were reduced significantly to between 0.21% and 12.98% (Table 3), effectively eliminating matrix effects and enhancing quantification accuracy.

#### 3.3.2. Linear Range

To address the limitations of UPLC-QE-Orbitrap-MS—such as limited dynamic range, signal saturation at high concentrations, and suppression of low-abundance analytes—this study systematically evaluated linear ranges on both UPLC-QE-Orbitrap-MS and UPLC-MS/MS platforms for quantifying diazepam and its metabolites in aquatic products.

Standard solutions were prepared in 0.2% formic acid–50% acetonitrile–water (*v*/*v*). Calibration curves were constructed by plotting the peak area ratio of analyte to internal standard (Y) against analyte concentration (X), followed by linear regression analysis. Both platforms showed excellent linearity over 0.2–200 ng/mL, with correlation coefficients (r^2^) > 0.997, confirming reliable quantification performance.

#### 3.3.3. Limits of Detection and Quantification

To determine the sensitivity of both mass spectrometry platforms, complex matrices from commonly consumed aquatic organisms (e.g., large yellow croaker, shrimp, scallop, squid, three-wart crab) were spiked with gradient concentrations of diazepam and its metabolites.

The limits of detection (LOD) and quantification (LOQ) were established based on signal-to-noise ratios: S/N ≥ 3 for LOD and S/N ≥ 10 for LOQ. Results showed that both UPLC-QE-Orbitrap-MS and UPLC-MS/MS could reliably detect diazepam, nordazepam, oxazepam, and temazepam at an LOD of 0.10 μg/kg and an LOQ of 0.20 μg/kg across all tested matrices. These findings demonstrate the method’s strong sensitivity and its applicability for trace-level detection in complex aquatic samples.

#### 3.3.4. Accuracy and Precision

**Figure 4:**
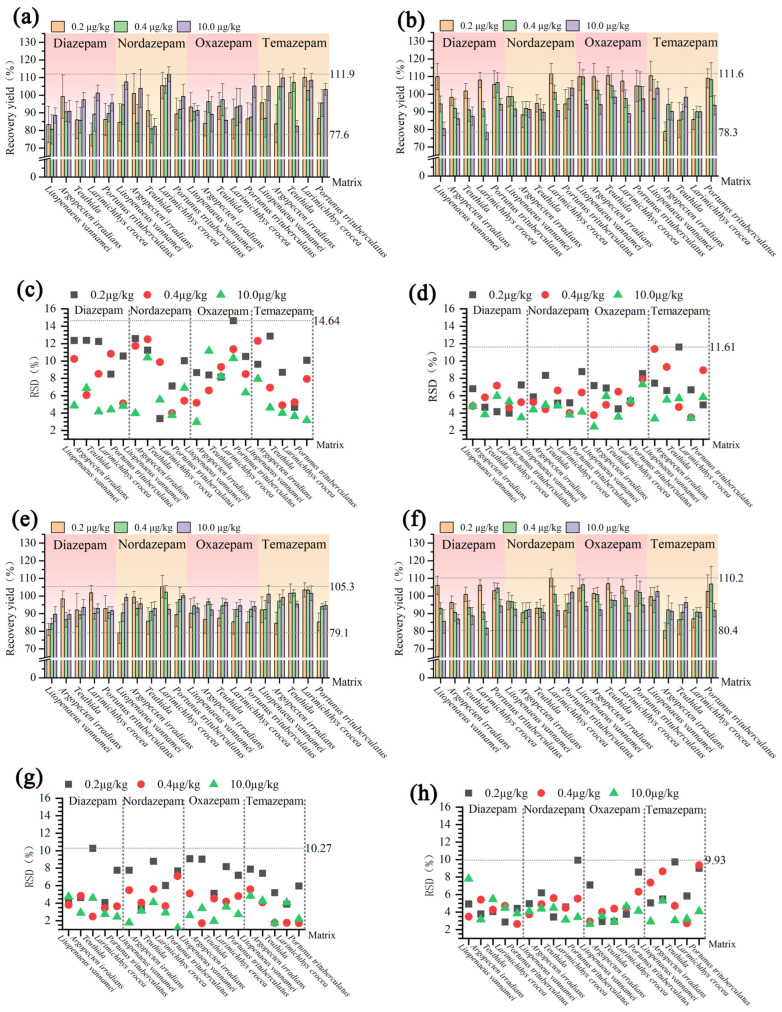
Recovery and precision for diazepam and metabolites in representative aquatic organisms determined on two analytical platforms. (**a**) Intra-batch recovery (UPLC-QE-Orbitrap-MS; *n* = 6; x ± SD). (**b**) Intra-batch recovery (UPLC-MS/MS; *n* = 6; x ± SD). (**c**) Intra-batch precision (UPLC-QE-Orbitrap-MS; *n* = 6). (**d**) Intra-batch precision (UPLC-MS/MS; *n* = 6). (**e**) Inter-batch recovery (UPLC-QE-Orbitrap-MS; *n* = 5; x ± SD). (**f**) Inter-batch recovery (UPLC-MS/MS; *n* = 5; x± SD). (**g**) Inter-batch precision (UPLC-QE-Orbitrap-MS; *n* = 5). (**h**) Inter-batch precision (UPLC-MS/MS; *n* = 5).

To assess the accuracy and precision of UPLC-QE-Orbitrap-MS and UPLC-MS/MS in detecting diazepam residues in aquatic organisms, five representative edible species—large yellow croaker (fish), shrimp (crustaceans), scallops (shellfish), squid (cephalopods), and three-wart crabs (crabs)—were selected. Blank samples from each were spiked at three concentrations (0.2, 0.4, and 10.0 μg/kg) for validation. Each concentration level was tested in sextuplicate, with three parallel groups assessed weekly over a period of five weeks. Results showed average intra-assay and inter-assay recoveries of 78.3–111.6% and 81.7–110.2%, respectively, with RSDs of 2.42–11.61% and 2.64–9.93%, respectively. An additional dataset yielded intra- and inter-assay recoveries of 7.6–111.9% and 79.1–105.3%, with RSDs of 2.97–14.64% and 1.16–10.27% (Figure 4). Both platforms achieved acceptable recoveries and precision, demonstrating stable accuracy and reproducibility in detecting diazepam and its metabolites across diverse aquatic matrices, making them suitable for real-sample analysis.

#### 3.3.5. Comparison of the Testing Capabilities of the Two Mass Spectrometry Platforms

To compare the reliability of UPLC-QE-Orbitrap-MS and UPLC-MS/MS, diazepam and its metabolites were spiked into various aquatic tissue matrices at three concentrations (0.2, 0.4, and 10.0 μg/kg). Method validation revealed relative deviation ranges between platforms of 0.11–19.13%, 0.12–18.01%, and 0.03–14.32% across the concentration levels. Repeatability and accuracy met FAO/WHO Codex Alimentarius Guidelines (CAC/GL-71) [31] for quantitative analysis. Pearson correlation coefficients were 0.994 (diazepam), 0.994 (nordazepam), 0.990 (oxazepam), and 0.990 (temazepam), all with *p* < 0.001, indicating strong agreement between platforms. Paired *t*-tests showed no statistically significant differences for any analyte (*p* > 0.001 or *p* > 0.05), confirming consistency between the two methodologies.

#### 3.3.6. Practical Sample Testing and Ecological Safety Applications

This method was applied to analyze 85 batches of aquatic organisms, including grass carp, common carp, silver carp, bighead carp, salmon, rainbow trout, sturgeon, catfish, perch, snakehead, black bass, croaker, crucian carp, shrimp, bay scallop, squid, and bullfrog. Concurrently, 150 environmental samples and 12 sediment samples were investigated for diazepam and its metabolites using this method. Results indicated diazepam residues were detected in 6 aquatic organism samples, with concentrations ranging from 0.288 to 2.569 μg/kg. The positive detection rate was 7.06%, indicating a risk of cumulative residues in animal-derived foods. Diazepam was detected in 5 aquatic environmental samples: 2 from natural rivers and 3 from artificial aquaculture ponds. Concentrations ranged from 7.25 to 271 ng/L in rivers and 0.783–3.16 μg/kg in aquaculture ponds. Diazepam levels in sediment correlated positively with those in water, consistent with findings by Huang et al. [32]. Diazepam and its metabolites resist natural degradation in the environment and readily accumulate across different media [33]. Their migration and transformation processes exhibit a dynamic equilibrium state, likely due to continuous material exchange between sediments and water bodies. The entire system involves complex transfer mechanisms among pollution sources, water bodies, sediments, and organisms, while the parent compound undergoes continuous biotransformation into metabolites. The interplay of multiple processes collectively forms a complex pollution system posing significant ecological risks. Therefore, establishing a rapid, multi-drug residue analysis method with high specificity, sensitivity, and reproducibility for detecting benzodiazepine residues in complex matrices is crucial for scientifically assessing pollution source risks and safeguarding public health. This represents a significant and current research priority.

## 4. Conclusions

This study developed a comprehensive matrix spectrum encompassing fish, crustaceans, shellfish, and cephalopods to establish a standardized LC-MS/MS pretreatment method for quantifying trace levels of diazepam and its active metabolites in aquatic products. The approach demonstrated high sensitivity, strong matrix interference resistance, and good repeatability. Both UPLC-QE-Orbitrap-MS and UPLC-MS/MS methods provided reliable trace-level detection in complex aquatic matrices, overcoming the limitations of single-platform detection and offering robust technical support for accurately assessing the ecological risks of diazepam contamination.

## Figures and Tables

**Table 1:** Chromatographic and high-resolution mass spectrometric parameters for diazepam and its metabolites determined by UPLC-QE-Orbitrap-MS.

Name	Chromatographic Retention Time (t, min)	Exact Mass (*m*/*z*)		Collision Energy (eV)	Scanning Method (ESI±)	Ionization Mode
		Precursor Ion (*m*/*z*)	Quantifier Ions (*m*/*z*)			
Diazepam	3.66	285.07892	193.08815	60	ESI+	[M + H]^+^
Nordazepam	3.48	271.06327	140.02582	60	ESI+	[M + H]^+^
Oxazepam	3.38	287.05818	241.05208	30	ESI+	[M + H]^+^
Temazepam	3.26	301.07383	255.06877	30	ESI+	[M + H]^+^
Diazepam-D5	3.65	290.11030	198.11949	60	ESI+	[M + H]^+^
Nordazepam-D5	3.48	276.09465	140.02583	60	ESI+	[M + H]^+^
Oxazepam-D5	3.38	292.08957	274.07840	30	ESI+	[M + H]^+^
Temazepam-D5	3.26	306.10522	260.09915	30	ESI+	[M + H]^+^

**Table 2:** MRM mass-spectrometric parameters for diazepam and its metabolites determined by UPLC-MS/MS.

Compound	Retention Time (min).	Precursor Ion (*m*/*z*).	Product Ions (*m*/*z*).	Declustering Voltage/eV	Collision Energy/eV
Diazepam	3.65	285.0	193.1 *	50	34.7
			153.9	50	34.2
Nordazepam	3.43	271.0	140.0 *	40	38.0
			165.0	40	37.0
Oxazepam+	3.64	287.0	240.9 *	50	30.0
			269.1	50	21.0
Temazepam	3.49	301.0	255.2 *	21	28.0
			283.0	21	18.3
Diazepam-D5	3.63	290.2	198.1	50	41.8
			154.1	50	35.8
Nordazepam-D5	3.41	276.1	213.0	60	37.2
			140.0	60	38.9
Oxazepam-D5	3.64	292.1	246.1	50	29.6
			227.1	50	47.0
Temazepam-D5	3.49	306.2	260.0	60	29.1
			288.0	60	47.0

* Denotes the quantifier ion.

**Table 3:** Regression equations, linear ranges, correlation coefficients, and matrix effects (ME) for diazepam and its metabolites.

Instrument	Compound of Interest	Regression Equations	Linear Range (ng/mL)	Correlation Coefficient (r^2^)	Absolute Value of ME (%)	
					External Standard Method	Internal Standard Method
UPLC-QE-Orbitrap-MS	Diazepam	Y = 0.3077X − 2.256 × 10^−2^	0.2~200	0.9993	18.68~51.32	5.71~10.13
	Nordazepam	Y = 0.4086X − 2.685 × 10^−2^	0.2~200	0.9983	11.57~69.36	3.85~9.62
	Oxazepam	Y = 0.3539X − 2.335 × 10^−2^	0.2~200	0.9971	15.48~59.67	1.75~8.64
	Temazepam	Y = 0.2766X − 8.201 × 10^−3^	0.2~200	0.9981	22.38~48.61	2.56~6.97
UPLC-MS/MS	Diazepam	Y = 0.269X + 9.00 × 10^−4^	0.2~200	0.9998	33.95~71.82	4.11~11.17
	Nordazepam	Y = 0.342X + 2.10 × 10^−4^	0.2~200	0.9988	17.51~70.75	1.68~11.68
	Oxazepam	Y = 0.171X − 3.16 × 10^−3^	0.2~200	0.9985	6.69~50.38	0.21~12.98
	Temazepam	Y = 0.285X + 4.57 × 10^−2^	0.2~200	0.9996	19.90~65.08	1.00~4.48

## Data Availability

The original contributions presented in the study are included in the article, further inquiries can be directed to the corresponding author.
